# Multilayer Perceptron-Based Wearable Exercise-Related Heart Rate Variability Predicts Anxiety and Depression in College Students

**DOI:** 10.3390/s24134203

**Published:** 2024-06-28

**Authors:** Xiongfeng Li, Limin Zou, Haojie Li

**Affiliations:** 1 Department of Physical Educantion, Xinzhou Normal University, Xinzhou 034000, China; 15513200878@xztu.edu.cn; 2 College of Physical Educantion, Jinggangshan University, Ji’an 343009, China; 3 School of Physical Educantion and Sports, Beijing Normal University, Beijing 100875, China; 202121070037@mail.bnu.edu.cn

**Keywords:** heart rate variability, anxiety, depression, college students, multilayer perceptron, exercise

## Abstract

(1) Background: This study aims to investigate the correlation between heart rate variability (HRV) during exercise and recovery periods and the levels of anxiety and depression among college students. Additionally, the study assesses the accuracy of a multilayer perceptron-based HRV analysis in predicting these emotional states. (2) Methods: A total of 845 healthy college students, aged between 18 and 22, participated in the study. Participants completed self-assessment scales for anxiety and depression (SAS and PHQ-9). HRV data were collected during exercise and for a 5-min period post-exercise. The multilayer perceptron neural network model, which included several branches with identical configurations, was employed for data processing. (3) Results: Through a 5-fold cross-validation approach, the average accuracy of HRV in predicting anxiety levels was 89.3% for no anxiety, 83.6% for mild anxiety, and 74.9% for moderate to severe anxiety. For depression levels, the average accuracy was 90.1% for no depression, 84.2% for mild depression, and 82.1% for moderate to severe depression. The predictive R-squared values for anxiety and depression scores were 0.62 and 0.41, respectively. (4) Conclusions: The study demonstrated that HRV during exercise and recovery in college students can effectively predict levels of anxiety and depression. However, the accuracy of score prediction requires further improvement. HRV related to exercise can serve as a non-invasive biomarker for assessing psychological health.

## 1. Introduction

Mental health problems faced by college students are a growing concern, and the problem of negative emotions among college students is becoming increasingly serious, with anxiety and depression becoming prevalent mental health challenges [1]. The college student population is particularly vulnerable to anxiety and depression due to academic stress [2], interpersonal relationships [3], and uncertain employment prospects [4]. The study of Faisal [5] pointed out that poor mental health status in higher education has become a serious public health problem globally [6]. The study of Faisal [6] found that mental health problems may lead to serious consequences, such as extreme behaviors like suicide. These studies emphasize the urgency and importance of mental health problems among college students.

HRV is the degree of variation between heartbeat intervals and is a physiological indicator of the flexibility of the autonomic nervous system to regulate cardiac rhythm [7]. The application of HRV in sports has become an area of great interest. Monitoring HRV during exercise can help athletes understand their physical condition, optimize their training programs, and improve their athletic performance [8,9]. Kirk [10] showed that HRV can be used to assess the physical adaptability and fatigue level of athletes. Through continuous monitoring of HRV, athletes and coaches can understand the effects of training on the body and adjust the training load in time to avoid overtraining and sports injuries. In addition, the study of Dewig [11] found that HRV also plays an important role in guiding recovery and adjusting training programs. The recovery phase after exercise is crucial for physical rehabilitation and adaptation. Monitoring HRV changes after exercise can help determine the appropriate rest and recovery time to ensure that the body is in optimal condition for the next round of training.

In this study, we focus on the activity of HRV variability during exercise to explore how heart rate variability during exercise affects physical and mental health. Many studies have shown that high HRV is often associated with better exercise adaptation and cardiovascular fitness. Levels of HRV may differ between exercise and resting states, which is closely related to the modulatory effects of exercise on the autonomic nervous system [12,13]. Important physiologic information can be provided by continuous monitoring of HRV during sports. For example, an increase in HRV may mean that the athlete is in a more relaxed state, which facilitates recovery and adaptation [14]. In contrast, a decrease in HRV may be a sign of overtraining or fatigue, requiring adjustments to the training program to avoid overexertion and potential injuries [15]. Studies have also found that the effect of exercise on HRV varies by individual and type of exercise. Some studies have shown that endurance training improves HRV, which may be related to adaptations in the cardiovascular system. In contrast, high-intensity training or overtraining may lead to a decrease in HRV, which indicates that the body is in a state of stress and needs adequate rest and rehabilitation [16]. In addition, advances in HRV monitoring technology have made its use in sports more popular and precise. Athletes can monitor HRV in real time through portable devices or apps to adjust the training intensity and periodization in time to obtain the best training effect and physical adaptation [17]. The research on the variability of exercise center rate is still deepening. Recent studies have shown that HRV is closely related to factors such as training load, recovery status, and psychological state. For example, some studies have found that emotional state has a significant effect on HRV, and a good psychological state can promote the recovery and stabilization of HRV [18]. These findings further emphasize the positive effects of HRV in exercise on mental health. Current research on HRV has not focused on changes during exercise, and there are no studies related to the prediction of mental health from HRV during exercise. However, with growing concerns about anxiety and depression, there is an urgent need to begin exploring the relationship between HRV and emotional states.

Multilayer perceptron is a deep learning model that is widely used in the field of machine learning. It learns complex features of data through multiple hidden layers and has good generalization ability [19]. Currently, multilayer perceptron has been successfully applied to image recognition, speech recognition, natural language processing, and other fields [20,21], and its potential in the field of sports has been gradually explored. On the one hand, traditional measures of negative emotions in college students mostly rely on the collection of subjective scales, which have certain subjectivity and limitations [22]. Consequently, this is the first time that multilayer perceptron has been applied in a prediction study for college students’ anxiety and depression. This study will utilize the multilayer perceptron algorithm combined with sensor data to explore its ability to predict negative emotions in college students, aiming to provide new research ideas and methods for college students’ mental health.

The objective of this study is to predict anxiety and depression in college students using sensor data and the multilayer perceptron algorithm. The innovation of this study is the application of the multilayer perceptron algorithm to the field of mental health in college students and the possibility of predicting anxiety and depression using exercise-related HRV data.

## 2. Participants and Methods

### 2.1. Participants

This study involved 845 university students between the ages of 18 and 22. Eligibility was contingent upon a demonstrated state of good health, with exclusions for severe medical conditions including, but not limited to, cardiovascular diseases, endocrine disorders, and documented psychiatric illnesses. Basic information on subjects (Table 1). Participants were also required to be free from any mobility or cognitive limitations that could affect their engagement in moderate-intensity physical activities. In accordance with the Declaration of Helsinki, the recruitment process was conducted in an ethically sound manner. Potential participants were provided with comprehensive information about the study’s objectives, potential risks, and potential benefits prior to providing written consent to participate. Subsequently, the research protocol underwent a comprehensive review by the Beijing Normal University’s Ethics Committee. The protocol was subsequently granted ethical approval (number: 20240115), thereby attesting to the study’s compliance with the most rigorous ethical standards pertaining to human subject participation.

### 2.2. Study Design

**Figure 1:**
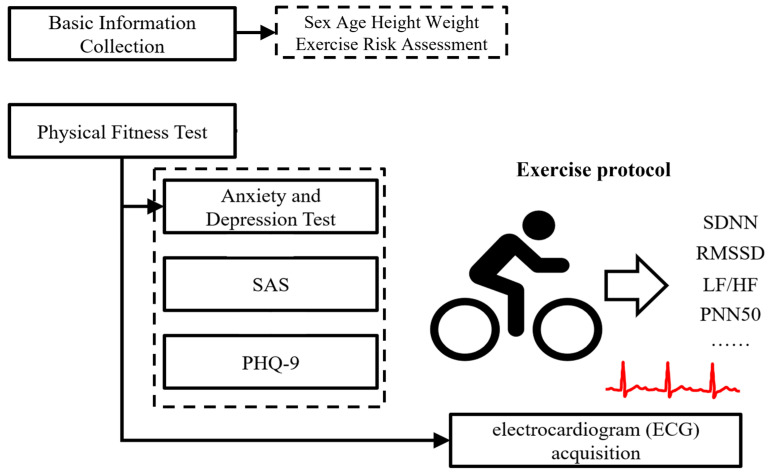
Flowchart of experimental design.

This research employed a cross-sectional design to assess the relationship between HRV during and after exercise and the presence of anxiety and depression among college students. Following the evaluation of anxiety and depressive symptoms, participants were given a 10-min rest period prior to the commencement of the exercise protocol to ensure a stable baseline. HRV data were collected during the exercise session and continued for a 5-min period post-exercise to capture the physiological recovery. The data collection was scheduled during two specific time frames each day, from 8:30 a.m. to 12:00 p.m. and from 1:00 p.m. to 3:00 p.m., in order to control for diurnal variations that might influence HRV measurements (Figure 1).

### 2.3. Cardiorespiratory Fitness Testing

The exercise intervention in this study was conducted using an ergometer bicycle dynamometer selected for its safety and adaptability for the participants. The height of the ergometer seat was individually adjusted for each participant based on their height, ensuring that the distance from the seat to the lowest point of the pedal stroke (bottom dead center) was equivalent to the distance from the greater trochanter of the femur to the plantar surface of the foot. This adjustment was crucial for maintaining optimal biomechanics and reducing the risk of injury during the exercise. The exercise workload was set at a moderate intensity, with a prescription of 75 watts for males and 50 watts for females. This approach is commonly employed to ensure an aerobic training stimulus while being considerate of the participants’ fitness levels. Prior to commencing the exercise, participants underwent a 5-min warm-up at a workload of 25 watts. This was done to gradually increase their heart rate and muscle temperature, thus preparing their bodies for the subsequent exercise bout. They were then instructed to maintain a cadence of approximately 60 revolutions per minute (RPM) over a 6-min period of cycling. This controlled cadence was chosen to elicit a consistent cardiorespiratory response. During the cycling exercise, participants were instructed to grip the handlebars firmly, maintain an upright posture, and keep their gaze forward. This was done to ensure consistency in body position across all participants, which is essential for obtaining reliable physiological data and for the subsequent analysis of HRV.

### 2.4. HRV Assessment

In this study, HRV was measured using the SmartCardio patch (Zhirou Cardio Patch, Zhejiang, China), a single-lead electrocardiogram (ECG) device affixed to the left chest of participants. This non-invasive approach facilitated the collection of HRV data during the exercise phase and continued into a five-minute recovery period post-exercise. The HRV parameters assessed included:

- Longest RR interval(s);
- Standard deviation of N-N intervals (SDNN) (ms);
- Root mean square of successive differences (RMSSD) (ms);
- Percentage of differences between adjacent NN intervals greater than 50 ms (PNN50) (%);
- HRV triangular index;
- Very-low-frequency (VLF);
- Low-frequency (LF);
- High-frequency (HF);
- LF/HF ratio.

These HRV indices provide a comprehensive assessment of cardiac autonomic modulation, reflecting the dynamic interplay between the sympathetic and parasympathetic branches of the autonomic nervous system. The selection of these indices was based on their established clinical relevance and their sensitivity to changes in the physiological state resulting from exercise and recovery.

The SmartCardio patch, known for its reliability and ease of use, allowed for the continuous and artifact-free recording of HRV data. The data acquisition spanned the exercise session and the subsequent recovery period, enabling a detailed analysis of the participants’ cardiac response to the physical challenge and their subsequent return to homeostasis.

The HRV analysis in this study was conducted in accordance with the guidelines set forth by the Task Force of the European Society of Cardiology and the North American Society of Pacing and Electrophysiology, thereby ensuring the validity and clinical significance of the findings.

### 2.5. Depression and Anxiety Assessment

The assessment of depression and anxiety was conducted using the Self-Rating Anxiety Scale (SAS) and the Patient Health Questionnaire-9 (PHQ-9), respectively.

The SAS comprises 20 items that assess various anxiety symptoms. Each item is rated on a 4-point scale, indicating the frequency of symptom occurrence, ranging from ‘not at all’ to ‘most of the time’. Raw scores are totaled and then multiplied by 1.25 to obtain standard scores. In this study, SAS standard scores were categorized as follows:

- Below 50: No anxiety symptoms;
- 50–59: Mild anxiety;
- 60 and above: Moderate to severe anxiety.

The PHQ-9 includes 9 items aligned with depression diagnostic criteria from the DSM-IV. Participants indicate the frequency of symptoms over the past two weeks on a scale from 0 to 3, with ‘not at all’ representing the lowest level of symptom occurrence and ‘nearly every day’ representing the highest. Total scores are calculated by summing individual item scores. Depression severity categories in this study were defined as follows:

- 0–4: No depression;
- 5–9: Mild depression;
- 10 and above: Moderate to severe depression.

Preparation for Assessment: Prior to the administration of the questionnaires, participants were provided with detailed instructions to ensure a comprehensive understanding of the assessment instruments, which is crucial for accurate self-reporting.

Mode of Administration: The assessments were administered in paper-and-pencil format, allowing participants to complete them at their own pace and ensuring uniform data collection.

The personnel involved in the assessment were trained research assistants who were blinded to the study hypotheses. They administered the questionnaires, provided clarification as needed, and ensured that the participants completed them independently.

### 2.6. Neural Network Architecture

The current study employs a branched neural network designed to address both regression and classification tasks, utilizing a common base architecture with task-specific output layers. This architecture processes input data in the form of 2D arrays. Both the HRV during the cycling period and the recovery period are treated as distinct dimensional features, simultaneously inputted into the network.

#### 2.6.1. Input and Preprocessing

The neural network receives input through a standardized input layer followed by a batch normalization layer to ensure data normalization.

#### 2.6.2. Branching Structure

Post normalization, the network diverges into four distinct branches. Each branch comprises the following components in sequence:

Fully Connected Layer: This layer consists of 116 neurons and applies a specific activation function determined through hyperparameter optimization.

Batch Normalization Layer: Standardizes the output of the previous layer, which aids in reducing internal covariate shift.

Dropout Layer: Introduces a dropout rate to prevent overfitting by randomly setting a fraction of input units to zero at each update during training.

Fully Connected Layer: Contains 32 neurons and utilizes the same activation function as the first fully connected layer within the branch.

#### 2.6.3. Activation Functions

Each branch employs one of the following activation functions: ELU, SELU, Sigmoid, or Tanh. The selection of these functions is based on hyperparameter optimization, aiming to enhance the network’s performance by leveraging the strengths of diverse activation functions.

#### 2.6.4. Branch Merging and Final Layers

The outputs of the four branches are concatenated into a single tensor. This merged output then passes through a final fully connected layer with 64 neurons and a ReLU activation function.

The final layer’s configuration is tailored to the task at hand: a single neuron is used in regression tasks for predicting a continuous target variable, while three neurons are employed in classification tasks to predict categorical outcomes.

#### 2.6.5. Batch Size

The choice of activation functions and batch size was found to significantly influence the network’s learning outcomes, highlighting the importance of these parameters in model training.

The network in this study was trained using a batch size of 4, which was determined to be the optimal size through hyperparameter tuning. This configuration provided a balance between convergence speed and model generalization.

#### 2.6.6. Hyperparameter Optimization

**Figure 2:**
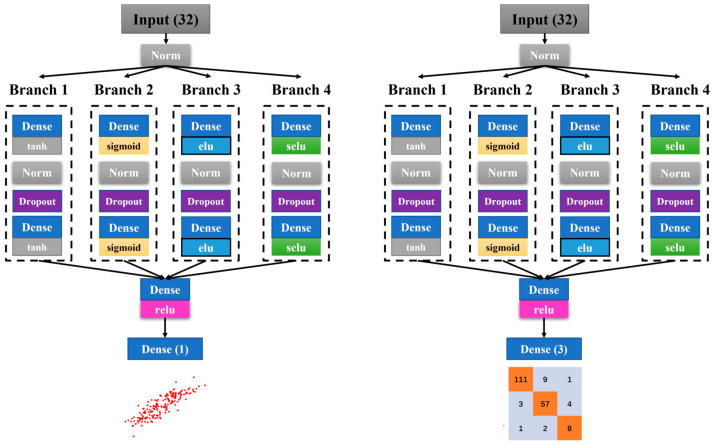
Multilayer Perceptron Network Architecture.

In this study, a pivotal component of the neural network design involved hyperparameter optimization through Optuna. Initially, the design framework was rudimentary, with the types of activation functions and the number of neurons in each branch being undetermined. The optimization process was configured with a total of 1000 trials, each training for 1000 epochs, where each trial explored different combinations of activation functions, neuron counts, and dropout rates. The criterion for optimal performance was the lowest loss on the test set. Ultimately, the network architecture was determined by the lowest loss, which specified distinct activation functions for each branch: ELU, SELU, Sigmoid, and Tanh. Thus, hyperparameter optimization became an integral part of the training process, effectively determining the activation functions (Figure 2).

### 2.7. Statistical Analysis

The models’ performance underwent rigorous evaluation through a 5-fold cross-validation methodology. This approach entailed dividing the dataset into five equal subsets, where each subset served as the test set while the remaining four constituted the training set. This iterative process was repeated five times, with each subset being utilized as the test set once.

For classification predictions, the anxiety and depression classifications generated five confusion matrices each, resulting in a total of ten confusion matrices. The model’s performance was evaluated by computing the average accuracy across these matrices, offering a comprehensive assessment of the classification task’s efficacy.

In the context of regression predictions for scoring, both anxiety and depression yielded five scatter plots each. These plots visually depicted the relationship between predicted scores and actual scores, facilitating a graphical assessment of the model’s predictive accuracy. The coefficient of determination (R^2^) was computed for each scatter plot, with the average R^2^ value serving as the metric for evaluating the model’s performance in the regression task. In order to validate the performance of the neural network in this study, the performance of support vector machines, random forests, and conventional MLP under classification and regression tasks were also compared.

## 3. Results

**Figure 3:**
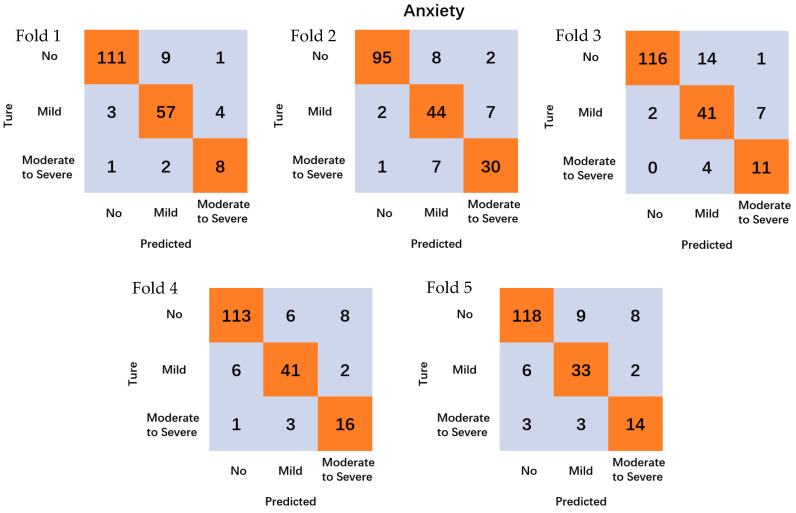
Confusion matrix for the prediction of anxiety by different folds.

The results of Table 2 and Figure 3 show the predictive accuracy of the HRV for anxiety levels of university students, with an average accuracy of 89.3% for non-anxiety levels, 83.6% for mild anxiety levels, and 74.9% for moderate to severe anxiety levels.

**Figure 4:**
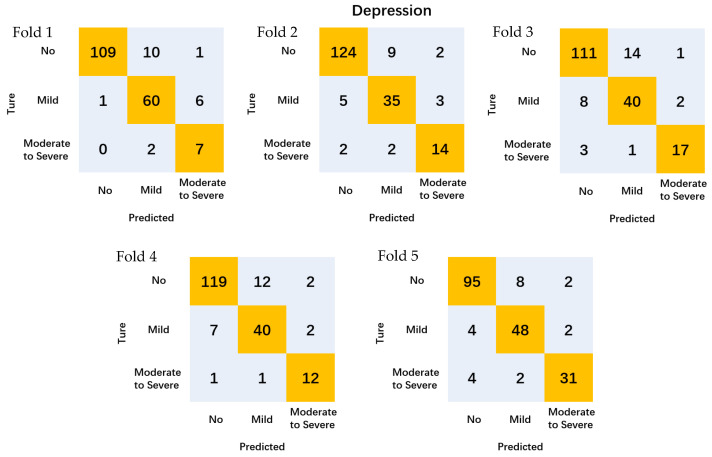
Confusion matrix for the prediction of depression by different folds.

The results in Table 3 and Figure 4 show the predictive accuracy of the HRV for depression levels in college students, with an average accuracy of 90.1% for non-depressed levels, 84.2% for mild depression levels, and 82.1% for moderate to severe depression levels.

**Figure 5:**
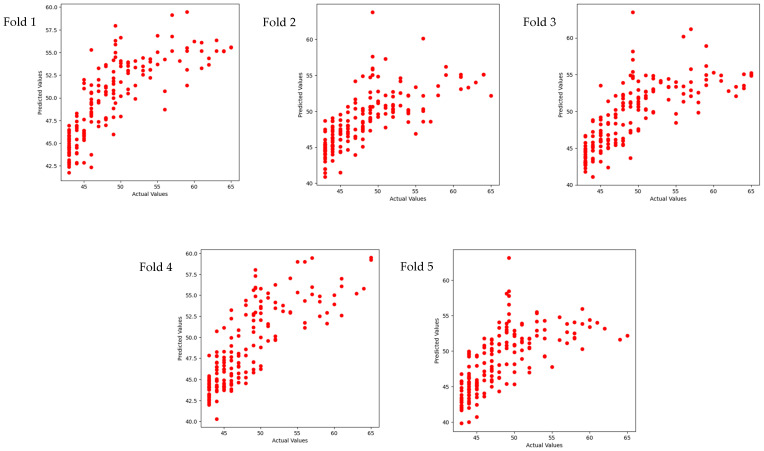
Scatterplot of five-fold cross-validation in a regression task for predicting anxiety scores.

**Figure 6:**
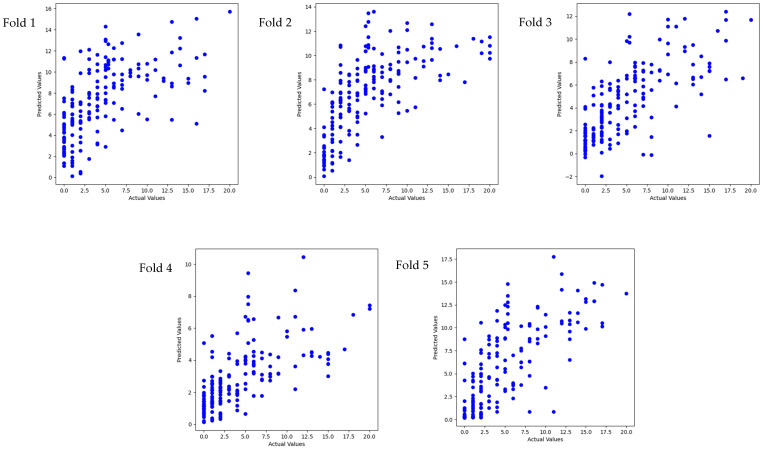
Scatterplot of five-fold cross-validation in a regression task for depression scores.

The results in Table 4, Figure 5 and Figure 6 show the predictive R-square of HRV for specific scores of anxiety and depression among college students, which reached a mean of 0.62 for anxiety and 0. 41 for depression.

The results in Table 5 show that compared to SVM, random forest, and MLP without branching structure, MLP with branching structure has the highest average accuracy in both regression and classification tasks.

SVM represents a support vector machine; MLP—No Branch represents a multilayer perceptron without any branching structure; MLP—With Branch represents a multilayer perceptron that incorporates a branching structure.

The MLP without a branching structure utilizes ReLU activation functions across all layers. It consists of four layers, each with 132 neurons, and maintains a total number of trainable parameters at approximately 50,000, which is equivalent to the MLP with a branching structure. The results in Table 5 show that compared to SVM, random forest, and MLP without branching structure, MLP with branching structure has the highest average accuracy in both regression and classification tasks.

## 4. Discussion

In this study, we observed a high accuracy of multilayer perceptron-based HRV in the prediction of anxiety and depression levels in college students. The prediction results for anxiety levels showed an average accuracy of 89.3% for non-anxiety states, 83.6% for mild anxiety levels, and 74.9% for moderate to severe anxiety levels. For depression levels, the prediction results showed a mean accuracy of 90.1% for non-depressed states, 84.2% for mild depression levels, and 82.1% for moderate to severe depression levels. In addition, the predicted R-square values of HRV for anxiety and depression in college students were 0.62 and 0.41, respectively. These results have important applications in practice. HRV, as a physiological indicator, is able to capture changes in the activity of the autonomic nervous system and is, therefore, considered a powerful indicator of an individual’s psychological state [23]. The HRV during exercise and the subsequent recovery period is a response to a specific physiological stress load, in comparison to the HRV observed during rest. Consequently, it can be postulated that the HRV during exercise and recovery may provide a more sensitive reflection of the regulatory capacity of the sympathetic/parasympathetic nervous system. In our study, through the high-accuracy prediction of exercise-related HRV, we were able to more accurately assess the anxiety and depression levels of college students, which provided a reliable scientific basis for mental health interventions. This means that we can detect and intervene in college students’ mental health problems earlier; in addition, the high prediction accuracy of HRV provides new possibilities for early intervention in college students’ mental health problems.

On the other hand, we observed that exercise-related HRV based on multilayer perceptron has higher accuracy in the prediction of anxiety level in college students, which implies that HRV can more accurately identify individuals with different levels of anxiety among college students, which can help to target interventions. Anxiety is a common mental health problem among college students, and timely intervention can effectively reduce anxiety symptoms and improve individuals’ quality of life and academic performance [24]. Therefore, the high accuracy of HRV as a non-invasive physiological indicator in identifying anxiety symptoms provides an effective tool for mental health professionals and healthcare teams to intervene early and support college students who may be facing anxiety distress. The results of our study also found that HRV was much more predictive of anxiety levels than depression levels in college students. This may be related to the differences in physiological mechanisms and psychological manifestations of anxiety and depression. This finding further confirms the research of Song [25], who found that although anxiety and depression are both common mental health problems, there are significant differences in their pathogenesis and presentation. Anxiety is often accompanied by physical reactions such as physical tension and rapid heartbeat, while depression is more often characterized by psychological symptoms such as low mood and loss of interest in daily activities [26]. The study in this paper found that the predictive ability of HRV for anxiety level of college students is much higher than that for depression level. This means that through HRV monitoring, we are more likely to capture possible anxiety problems among college students and thus can provide more timely intervention and support. Therefore, placing more emphasis on the use of exercise-related HRV in anxiety identification in the college student population is expected to provide a more accurate and effective means of mental health intervention.

In addition, timely mental health interventions are not only critical to the personal quality of life of college students, but also have a positive impact on their academic performance. Several studies have shown that anxiety and depressive symptoms can negatively affect college students’ academic ability and performance [27,28]. Therefore, timely detection of mental health problems through exercise-related HRV is expected to improve the academic performance of college students, thereby contributing to their personal development and future career success.

Overall, the results of this study provide an important scientific basis for the prevention and intervention of mental health problems in college students. With the predictive accuracy of exercise-related HRV, we can more accurately assess the anxiety and depression levels of college students and provide a scientific basis for mental health intervention. This helps to detect and intervene in the mental health problems of college students at an early stage and improve their quality of life and academic performance [29]. Further analysis of these findings suggests that exercise-related HRV can be used as an effective indicator for predicting mental health status. The significant association between HRV and anxiety and depression symptoms suggests that HRV is not only a physiological indicator but can also reflect changes in psychological status, thus providing a new perspective for the assessment of mental health problems.

### 4.1. Strengths

The article employs a multilayer perceptron-based method for analyzing exercise-related HRV, which represents an innovative attempt to predict anxiety and depression levels in college students. The method combines machine learning algorithms and physiological metrics to provide a new way to study the mental health of college students.

### 4.2. Limitations

The study may have been limited by sample size and sample representativeness, which may have affected the ability to generalize the results. A larger and more diverse sample may have contributed to the reliability and applicability of the study. In addition, machine learning algorithms, while excellent at prediction, have low interpretability of results. Understanding the underlying physiological mechanisms and influencing factors is crucial to further optimize prediction models and guide practice.

## 5. Conclusions

In conclusion, our study demonstrates the potential of employing multilayer perceptron-based exercise-related HRV analyses as a novel tool for predicting anxiety and depression levels in college students. The findings indicate that exercise-related HRV can effectively discriminate between different levels of anxiety and depression with significant predictive accuracy.

The combination of exercise-related HRV and machine learning algorithms represents a sensitive predictor of at-risk populations, enabling mental health professionals to intervene accordingly and promote mental health among college students.

## Figures and Tables

**Table 1:** Characteristics of participants.

	Female	Male
Age (years)	19.25 ± 1.49	20.25 ± 1.58
Height (cm)	166.07 ± 2.46	174.02 ± 3.92
Weight (kg)	53.23 ± 5.94	71.11 ± 6.83
Body mass index (kg/m^2^)	19.61 ± 2.07	25.03 ± 3.03
Self-Rating Anxiety Scale	41.76 ± 16.28	40.58 ± 18.65
Patient Health Questionnaire	7.23 ± 5.86	8.66 ± 6.58
Longest RR Interval (s)	1.02 ± 0.39	1.20 ± 0.68
SDANN	99.53 ± 30.75	97.54 ± 30.73
RMSSD	13.56 ± 6.52	16.44 ± 7.47

Note: Data are expressed as mean ± standard deviation.

**Table 2:** Predictive accuracy of different folds for anxiety.

Anxiety			Accuracy			
Levels	Fold 1	Fold 2	Fold 3	Fold 4	Fold 5	Average
No	91.7%	90.4%	88.5%	88.9%	87.4%	89.3%
Mild	89.1%	83.1%	82.0%	83.6%	80.4%	83.6%
Moderate to Severe	72.7%	78.9%	73.3%	80%	70.0%	74.9%

**Table 3:** Predictive accuracy of different folds for depression.

Depression			Accuracy			
Levels	Fold 1	Fold 2	Fold 3	Fold 4	Fold 5	Average
No	90.8%	91.8%	88.4%	89.0%	90.4%	90.1%
Mild	89.5%	81.3%	80.0%	81.6%	88.8%	84.2%
Moderate to Severe	77.0%	77.7%	85.0%	86.7%	83.7%	82.1%

**Table 4:** R-square for the prediction of anxiety and depression by different folding.

			R^2^			
	Fold 1	Fold 2	Fold 3	Fold 4	Fold 5	Average
Anxiety	0.75	0.64	0.63	0.54	0.52	0.62
Depression	0.33	0.23	0.48	0.59	0.44	0.41

**Table 5:** Comparison of prediction accuracy between MLP and other models.

	Anxiety		Depression	
	R^2^	Accuracy	R^2^	Accuracy
	Score Prediction	Classifications	Score Prediction	Classifications
SVM	0.45	64.3%	0.14	60.4%
Random Forest	0.47	81.1%	0.37	57.4%
MLP—No Branch	0.59	73.4%	0.23	72.3%
MLP—With Branch	0.62	82.6%	0.41	85.47%

Note: R^2^ and accuracy for regression and classification tasks for all models are the average values after 5-fold cross-validation.

## Data Availability

The data that support the findings of this study are available on request from the corresponding author.
